# Post-Game High Protein Intake May Improve Recovery of Football-Specific Performance during a Congested Game Fixture: Results from the PRO-FOOTBALL Study

**DOI:** 10.3390/nu10040494

**Published:** 2018-04-16

**Authors:** Athanasios Poulios, Ioannis G. Fatouros, Magni Mohr, Dimitrios Draganidis, Chariklia K. Deli, Konstantinos Papanikolaou, Apostolos Sovatzidis, Theofano Nakopoulou, Georgios Ermidis, Theofanis Tzatzakis, Vasiliki C. Laschou, Kalliopi Georgakouli, Agisilaos Koulouris, Panagiotis Tsimeas, Athanasios Chatzinikolaou, Leonidas G. Karagounis, Dimitrios Batsilas, Peter Krustrup, Athanasios Z. Jamurtas

**Affiliations:** 1 School of Physical Education and Sport Science, University of Thessaly, Karies, 42100 Trikala, Greece; athanpoul@gmail.com (A.P.); fatouros@otenet.gr (I.G.F.); dimidraganidis@gmail.com (D.D.); delixar@pe.uth.gr (C.K.D.); guspapa93@gmail.com (K.P.); nakopoyloy@hotmail.com (T.N.); tzatzakis23@gmail.com (T.T.); lavassia123@gmail.com (V.L.); kgeorgakouli@gmail.com (K.G.); agisilaoskoulouris@gmail.com (A.K.); ptsimeas@gmail.com (P.T.); batsilas2000@yahoo.gr (D.B.); 2 Faculty of Natural and Health Sciences, University of the Faroe Islands, Jónas Broncksgøta 25, 3rd Floor, Tórshavn 100, Faroe Islands; magnim@setur.fo; 3 Center of Health and Human Performance, Department of Food and Nutrition, and Sports Science, University of Gothenburg, 411 20 Gothenburg, Sweden; 4 Department of Sports Science and Clinical Biomechanics, SDU Sport and Health Sciences Cluster (SHSC), University of Southern Denmark, 5230 Odense, Denmark; pkrustrup@health.sdu.dk; 5 Surgery Department, General Hospital of Thessaloniki “Agios Dimitrios”, 546 34 Thessaloniki, Greece; t.sovatzidis@gmail.com; 6 Dipartimento di Scienze Motorie e del Benessere, Università degli Studi di Napoli “Parthenope”, 80133 Napoli, Italy; germidis1990@gmail.com; 7 School of Physical Education and Sports Science, Democritus University of Thrace, 69100 Komotini, Greece; athchatz.tefaa@gmail.com; 8 Institute of Nutritional Science, Nestlé Research Centre, 1015 Lausanne, Switzerland; leonidas.karagounis@rdls.nestle.com; 9 Experimental Myology and Integrative Physiology Cluster, Plymouth Marjon University, Plymouth PL6 8BH, UK; 10 Sport and Health Sciences, College of Life and Environmental Sciences, University of Exeter, Exeter EX4 4SB, UK

**Keywords:** protein supplementation, football, in-season, field activity, performance, congested fixture

## Abstract

The effects of protein supplementation on performance recovery and inflammatory responses during a simulated one-week in-season microcycle with two games (G1, G2) performed three days apart were examined. Twenty football players participated in two trials, receiving either milk protein concentrate (1.15 and 0.26 g/kg on game and training days, respectively) (PRO) or an energy-matched placebo (1.37 and 0.31 g/kg of carbohydrate on game and training days, respectively) (PLA) according to a randomized, repeated-measures, crossover, double-blind design. Each trial included two games and four daily practices. Speed, jump height, isokinetic peak torque, and muscle soreness of knee flexors (KF) and extensors (KE) were measured before G1 and daily thereafter for six days. Blood was drawn before G1 and daily thereafter. Football-specific locomotor activity and heart rate were monitored using GPS technology during games and practices. The two games resulted in reduced speed (by 3–17%), strength of knee flexors (by 12–23%), and jumping performance (by 3–10%) throughout recovery, in both trials. Average heart rate and total distance covered during games remained unchanged in PRO but not in PLA. Moreover, PRO resulted in a change of smaller magnitude in high-intensity running at the end of G2 (75–90 min vs. 0–15 min) compared to PLA (P = 0.012). KE concentric strength demonstrated a more prolonged decline in PLA (days 1 and 2 after G1, P = 0.014–0.018; days 1, 2 and 3 after G2, P = 0.016–0.037) compared to PRO (days 1 after G1, P = 0.013; days 1 and 2 after G2, P = 0.014–0.033) following both games. KF eccentric strength decreased throughout recovery after G1 (PLA: P=0.001–0.047—PRO: P =0.004–0.22) in both trials, whereas after G2 it declined throughout recovery in PLA (P = 0.000–0.013) but only during the first two days (P = 0.000–0.014) in PRO. No treatment effect was observed for delayed onset of muscle soreness, leukocyte counts, and creatine kinase activity. PRO resulted in a faster recovery of protein and lipid peroxidation markers after both games. Reduced glutathione demonstrated a more short-lived reduction after G2 in PRO compared to PLA. In summary, these results provide evidence that protein feeding may more efficiently restore football-specific performance and strength and provide antioxidant protection during a congested game fixture.

## 1. Introduction

Football (soccer) is a high-intensity intermittent-type team sport characterized by varied movement patterns and an exceptionally high frequency of activity changes [1]. During a game, players usually cover ≥10 km, with high-intensity running and sprinting accounting for 2–3 km and ~0.5 km, respectively [2,3]. In general, football game-play is very demanding, with mean heart rate reaching values ≥80% of maximal heart rate [4,5,6]. The majority of explosive actions during a game are ballistic-type movements incorporating a strong eccentric component such as running, accelerating, decelerating, changes of direction, tackling, jumping, and shooting [7,8]. When these movements are performed intensely, as during a football game, they may induce microtrauma of muscle fibre, which could result in performance deterioration [9,10,11,12,13]. High-intensity running, repeated sprint ability, jumping potential, and strength are considerably impaired during the final minutes of a game and during recovery [2,9,11,14,15,16,17,18,19,20].

Skeletal muscle injury due to exercise-induced muscle damage (EIMD) is associated with increased proteolysis and protein breakdown [21], tissue disruption at the membrane and subcellular level (sarcomeric and non sarcomeric compartments), and the release of pro-inflammatory cytokines (e.g., inteleukins IL-1 and IL-6) by muscle and other tissues to mobilize local and systemic immune reserves [21,22]. Infiltrating white blood cells (WBC), i.e., neutrophils first and macrophages later, produce and release reactive oxygen species (ROS) via the NADPH oxidase complex, a process also called respiratory burst [23,24]. This may cause an upregulation of the activity of antioxidant systems and, as such, redox status perturbations, and may lead to a secondary damage to non-injured muscle tissue [23,24]. During the respiratory burst, oxidative stress and antioxidant status markers, e.g., thiobarbituric acid reactive substances (TBARS), protein carbonyls (PC), reduced glutathione (GSH), and total antioxidant capacity (TAC), are profoundly altered in various cellular compartments or tissues and in the circulation [25]. A second healing/repair phase follows this first pro-inflammatory phase, which is associated with satellite cell activation and elevated protein synthesis in skeletal muscle myofibre [26]. The characteristics (intensity, duration, and eccentric component) of the preceding exercise stimulus usually determine the time of onset and total duration of this second phase during which performance usually recovers [13]. A football game induces EIMD and an acute inflammatory response that leads to performance deterioration for as long as 24–72 h, depending on the player’s activity profile during the game [10,12,27]. In fact, when football players are exposed to demanding game/training schedules, it appears that they are under considerable physiological stress, as evidenced by a prolonged increase in several leukocyte subpopulations [28]. A football season usually comprises 50–90 games for a player, and in numerous professional leagues it is common for players to participate in games performed 3–4 days apart. Such congested schedules allow only a limited time for optimal recovery between consecutive games, which may be inadequate to eliminate fatigue and restore skeletal muscle homeostasis and performance, rendering athletes susceptible to injuries [9,29,30].

Several approaches are utilized to alleviate EIMD and inflammatory manifestations, enhance muscle healing, attenuate performance deterioration, and accelerate recovery [31]. These different recovery strategies may be categorized as rehabilitation and physical therapy methods (e.g., cryotherapy, compressive loading techniques, etc.), pharmacological (e.g., anti-inflammatory agents, ACE inhibitors, phosphodiesterase inhibitors etc.), exercise-related treatments (e.g., stretching), and nutritional approaches/supplements (e.g., anti-antioxidants, herbals, ω-3-fatty acids, β-hydroxy-β-methylbutyrate, etc.) [13,32]. These interventions aim to attenuate performance deterioration during the EIMD-associated inflammatory response on one hand and accelerate muscle and tendon healing during the regeneration phase on the other, to allow timely performance restoration [13]. However, currently there is no general consensus on EIMD treatment and performance recovery. In contrast, several reports suggest that disruption of the inflammatory response may hinder training adaptations and, ultimately, recovery [21,33].

Certain nutritional recovery interventions suggest that increased protein and carbohydrate intake may mediate regeneration processes and promote muscle regeneration and, in so doing, accelerate recovery of performance [34]. Protein supplementation has been shown to expedite skeletal muscle protein turnover by interfering with its synthesis and degradation under conditions of increased physiological stress that favor a negative protein balance such as those applied during a congested football fixture [35,36,37,38,39]. However, it has been argued that protein administration may not improve EIMD symptoms and promote performance recovery despite an acute upregulation of protein synthesis during recovery from intense exercise [35]. Although football is the most popular sport worldwide and has a specific game/training activity profile that is related to a unique phenotype of EIMD and recovery [4,5,9,10,12,27], there is no information on the effects of protein supplementation on recovery kinetics during a congested schedule. So far, studies have investigated the interaction between protein ingestion (or its co-ingestion with carbohydrates) and football-associated inflammatory response and recovery in response to a 4-day intense training period [40], a single competitive game [41], and tests simulating a football game [42,43,44]. These studies report that although an increased total protein intake may attenuate the rise of EIMD markers, it may not affect the inflammatory ones (e.g., cytokines). These studies are highly inconclusive regarding the effects of protein supplementation on the performance recovery of football players following various modes of intervention [40,41,42,43,44]. Moreover, it appears that a 70-min intermittent-type exercise of variable intensity that resembles team-sport play as in football may augment daily protein requirements above current recommended dietary allowances (RDA) levels (0.8–1.0 g/kg/day) [45]. It would be plausible, then, to assume that football activity in real-life competition performed according to a congested game fixture may increase further daily protein requirements for optimal recovery. On the other hand, increased carbohydrate intake appears not to affect performance recovery after football activity despite an attenuation of associated EIMD responses [41,44]. This study was therefore designed to investigate whether increased protein intake affects inflammatory and performance recovery kinetics in response to repeated football games performed only 3 days apart. Based on previous literature on the effects of protein feeding on exercise performance, we hypothesized that increased post-game protein feeding would promote performance and attenuate inflammatory, oxidative stress and muscle damage responses during recovery after two football games performed 3 days apart.

## 2. Materials and Methods

### 2.1. Experimental Design

**Figure 1:**
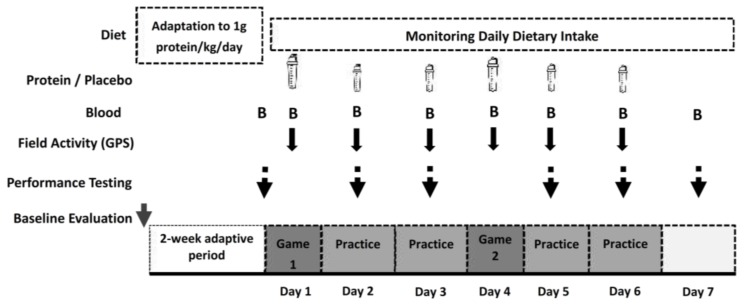
Experimental flowchart.

**Figure 2:**
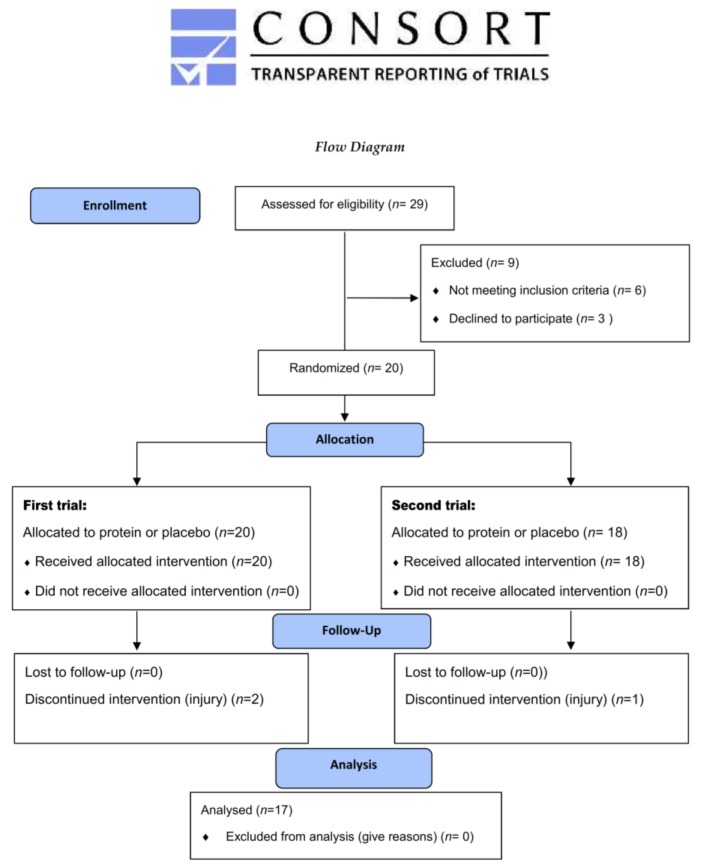
CONSORT 2010 flow diagram.

Figure 1 illustrates the experimental flowchart of the study and Figure 2 shows the CONSORT diagram of the study. A randomized, two-trial (placebo vs. protein consumption), cross-over, double-blind, repeated measures design was applied. The study was performed 5 days after completion of the participants’ in-season. Prior to each trial, participants had their body mass, height, resting metabolic rate (RMR), body composition, and daily dietary intake measured. Based on a dietary analysis, participants were given a dietary plan (taking into account the RMR and total daily physical activity related energy expenditure), providing a standard protein intake of 1 g protein/kg/day over the initial 2-week adaptive period. This protein intake is accepted as the average and population-safe protein intake during periods of very low physical activity and/or exercise levels [46,47]. During this adaptive period, volunteers were also familiarized with experimental procedures and participated in very light training (at a local football facility) aimed at developing game tactics and team cohesion (only before the first trial). At the end of the adaptation period and before each experimental trial, participants underwent a 3-day baseline performance testing at University facilities.

Each experimental trial included administration of either protein (PRO trial) or placebo (PLA trial). Each trial included two 90-min games (G1/G2) performed 3 days apart (days 1 and 4 at a local pitch, as a simulation of a typical in-season week with G1 performed on Sunday and G2 on Wednesday), two practice and testing sessions in between games (days 2 and 3), and two practices (days 5 and 6) and three testing sessions (days 5, 6 and 7) after G2. Practices during the second day after each match (Tuesday and Friday) were characterized by increased intensity and volume compared to the one performed during the first day (Monday and Thursday). Investigators designed and implemented all practice sessions, which followed the training schedule usually adopted by professional football teams during periods with games performed every 3 days. Volunteers were randomly assigned to two teams (equally representing all field positions) that played against each other in the two games (organized according to official regulations) performed during each trial (players participated in full 90-min games, i.e., there were no substitutions). On game days, volunteers participated only in morning testing sessions but not practices. A warm-up and a cool-down period were performed before and after each game, respectively. Scouts and coaches from professional clubs attended all games to increase the participants’ motivation and competitiveness to a level corresponding to that of a formal competition. Games were performed at 15.00 under normal conditions (20–23 °C, humidity ~60%). During the games, participants consumed only water ad libitum. Field activity during games and practices was recorded using high time-resolution GPS instrumentation and heart-rate monitoring. Prior to each game, a standard breakfast and meal was consumed by all players as previously described [4,9]. A 3-week washout period was adapted between trials. Diet intake was monitored daily during both trials and the wash-out period. During the two experimental trials, participants followed the same dietary protocol consisting of adequate amounts of protein and carbohydrate intake both on game (carbohydrate: ~6.7 g/kg—protein: ~1.3 g/kg) and training days (carbohydrate: ~5.1 g/kg—protein: ~1.2 g/kg). Current nutritional recommendations for athletes engaged in moderate amounts of training suggest a protein and carbohydrate consumption of 1.0–1.5 and 4–6 g/kg/day, respectively [46]. Moreover, it has been recently shown that intermittent-type activity of similar nature, intensity, and duration as football activity used in this study results in an estimated average intake requirement of 1.20–1.40 g/kg/day [45], which coincides with the daily protein intake levels used in this study.

Blood samples were collected before G1 of each trial and then daily for 7 consecutive days. All testing and blood sampling sessions were performed at the University laboratory at the same time of day in both trials to control for the effect of circadian variations.

### 2.2. Participants

A preliminary power analysis (effect size > 0.55, probability error of 0.05, power of 0.90), indicated that a sample size of 16–18 subjects is necessary to detect a statistically meaningful treatment effect among serial measurements in response to repeated football games [9,48]. Consequently, 29 competitive male football players were initially approached/interviewed, but 20 participated in the study. Participants’ characteristics at baseline are shown in Table 1. Participation in the study was secured if volunteers (1) had played at a competitive level (top three divisions) for ≥4 years; (2) were free of any recent history of illnesses, musculoskeletal problems, and metabolic diseases; (3) had not used any supplements and medications (for ≤6 months prior to the study); (4) were non-smokers; and (5) participated in ≥5 training sessions/week and played ≥1 game/week. All volunteers signed an informed consent form after they were fully informed about all benefits, risks, and discomforts of this investigation. All procedures were applied in accordance with the 1975 Declaration of Helsinki, as revised in 2000, and approval was obtained from the Ethics Committee of School of Physical Education and Sport Science, University of Thessaly (1078/2-2/10-2-2016). The study is registered at ClinicalTrials.gov (identifier: NCT03348267).

### 2.3. Supplementation Protocol

Participants consumed either a protein concentrate or a placebo in a random order. On game days, participants consumed 80 g of milk protein concentrate (Milk Protein Smooth, My Protein, UK) to increase total protein intake or an isoenergetic placebo (maltodextrin) that increased total carbohydrate intake over a 6 h recovery period starting immediately post-match (Table 2). Drinks were consumed as repeated “pulsed” doses, with the first dose administered immediately after the game and then one every 3 h on two occasions (+3 h, +6 h). The total amount of protein (80 g) was based on a previously reported intermediate feeding pattern [49,50], with the difference that three doses were used (25 g after game, 30 g at +3 h, and 25 g at +6 h) instead of four because a shorter recovery time interceded between the end of the game and night’s sleep (game ended at 6:00 pm). On each training day, participants consumed either a protein drink (20 g of protein) or a placebo (maltodextrin) with breakfast. The 25 g, 30 g, and 20 g servings of protein supplement contained 25 g of protein (80% casein & 20% whey)/4.7 g carbohydrate/1.6 g fat/~133 kcals, 30 g of protein (80% casein & 20% whey)/5.6 g carbohydrate/1.9 g fat/~160 kcals, and 20 g of protein (80% casein & 20% whey)/3.75 g carbohydrate/0.25 g fat/~97 kcals, respectively. The protein supplement did not contain any antioxidant ingredients (e.g., vitamins C or E, zinc, selenium, etc.). The supplementation protocol resulted in a high total carbohydrate intake in PLA and a high total protein intake in PRO on match days, especially during the post-match period (Table 2). Due to potential bias attributed to a placebo effect, participants were completely blinded to the supplement condition. All drinks were isovolumetric (~400 mL), consumed along with water, and flavored with banana to make the contents indistinguishable and non-transparent. For the protein trial, participants were asked 12 times each (once per day) whether they realized if the drink was the experimental supplement or placebo. Out of a total of 204 responses (17 players/trial), 96 times responded “I do not know”, 62 times guessed incorrectly, and 46 times guessed correctly (probably due to chance). Only 2 of 17 participants guessed correctly both PRO and PLA supplements. Therefore, we tend to believe that the players were well-blinded, and, as such, the placebo effect was eliminated. In both trials, participants were instructed to follow a daily nutrition pattern consisting on three main meals (breakfast, pre-game or pre-training lunch, and post-game or post-training dinner), as well as two snacks (before lunch and post-game or post-training). Protein intake in breakfast was derived from dairy and eggs, while that in lunch and dinner was based on various sources of animal-based proteins (poultry, egg, and meat). Protein intake in the two snacks was based on nuts and dairy. Participants were given various food equivalents (from sources described here) by a trained dietitian to provide the required protein intakes for game and training days.

### 2.4. Diet Monitoring

Participants were instructed by a registered dietitian how to record food/fluid servings and sizes and completed 7-day diet recalls evaluating their daily nutrient and energy intake during both trials and the washout period to ensure that during the second trial they maintained the same dietary pattern that used during the first trial. Diet recalls were analyzed using the Science Fit Diet 200 A (Science Technologies, Athens, Greece).

### 2.5. Measurement of Game/Practice Locomotor Activity Pattern

Field locomotor activity during game-play and practices was monitored using high time resolution global positioning system (GPS, 15 Hz with 100 Hz triaxial accelerometry; GPSport, Canberra, Australia) instrumentation as previously described [9,10]. Field activity was classified as total distance covered during a game, distance covered with running at speeds >14 km/h, high intensity running (HIR, distance covered at speeds >19 km/h), number of accelerations (1–2 m/s, 2–3 m/s, >3 m/s), and number of decelerations (1–2 m/s, 2–3 m/s, >3 m/s). HIR was also recorded for each 15-min period for each game (0–15 min, 15–30 min, 30–45 min, 45–60 min, 60–75 min, and 75–90 min), and the % decline of HIR during the last 15-min intervals of each half (30–45 min and 75–90 min) compared to the first 15-min period of each game was calculated as a measure of fatigue. Data were analyzed for each 5-min period of each game in order to detect the 5-min period with the highest HIR performance for each half of each game (peak 5-min). Furthermore, HIR was recorded for the next 5-min period immediately after the peak 5-min period in each half of each game (next 5-min period). Intensity during game-play and practices was monitored using continuous heart rate measurements (Team Polar, Polar Electro Oy, Kempele, Finland).

### 2.6. Descriptives

Body mass and height were measured on a beam balance with a stadiometer (Beam Balance-Stadiometer, SECA, Vogel & Halke, Hamburg, Germany) as previously described [4]. Body mass index was calculated as mass per height squared. Dual-emission X-ray absorptiometry (GE Healthcare, Lunar DPX-NT) was used for body composition assessment as previously published [51]. Open-circuit spirometry was utilized for assessment of maximal oxygen consumption (VO_2max_) using an automated online pulmonary gas exchange system (Vmax Encore 29, BEBJO296, Yorba Linda, CA, USA) via breath-by-breath analysis during a graded exercise testing on a treadmill (Stex 8025 T, Korea) as previously described [9]. For RMR assessment, resting VO_2_/CO_2_ was measured in the morning (07.00–09.00) after an overnight fast using an open-circuit indirect calorimeter with a ventilated hood system (Vmax Encore 29, BEBJO296, Yorba Linda, CA, USA), and the 24-h RMR was calculated as previously described [52]. Football-specific conditioning was measured using the Yo-Yo intermittent endurance test 2 (Yo-Yo IE2) and the Yo-Yo intermittent recovery test 2 (Yo-Yo IR2) with procedures previously described [53]. Yo-Yo and VO_2max_ testing were performed on separate days.

### 2.7. Performance Measurements

Players’ time to complete a 10-m and 30-m sprint was recorded by infrared photocells with a precision of 0.01 seconds (Newtest, Finland) as described [21]. Countermovement jump height (CMJ) was measured on an Ergojump contact platform (Newtest, Finland) as previously described [4]. Concentric and eccentric isokinetic peak torque of the knee extensors (KE) and knee flexors (KF), respectively, in both limbs was measured on an isokinetic dynamometer (Cybex 770, USA) at 120 °/s as previously described [10]. Delayed onset of muscle soreness (DOMS) in the knee flexors (KF) and extensors (KE) of both limbs was evaluated as previously described [9].

### 2.8. Blood Sampling

Fasting blood samples were collected by venipuncture using a disposable needle (20-gauge) and a Vacutainer tube holder from an antecubital arm vein with the participants sitting. Plasma was prepared by centrifugation (1370 g, 4 °C, 10 min) from blood samples collected into tubes containing ethylenediaminetetraacetic acid (EDTA) to measure creatine kinase activity (CK) and TBARS. Serum (to measure TAC) was prepared by centrifugation (1370 g, 4 °C, 10 min) from blood samples containing SST-Gel/clot activator that were first allowed to clot at room temperature. Packed erythrocytes (RBC) were prepared after lysis of plasma samples [54] to measure PC, GSH, and haemoglobin (Hb). All samples were stored at −75 °C to −80 °C in multiple aliquots until assayed. A small portion (2 mL) of whole blood was collected in tubes containing ethylenediaminetetraacetic acid to assess WBC, Hb, and hematocrit using an automated hematology analyser (Mythic 18, Orphee SA, Geneva, Switzerland).

All samples were thawed only once before being analyzed and were protected from light and auto-oxidation. All assays were performed in duplicate, and samples collected after a game were corrected for plasma volume changes as described [55].

CK was measured using a Clinical Chemistry Analyzer Z 1145 (Zafiropoulos Diagnostica, Greece) with commercially available kits (Zafiropoulos, Greece). For measuring PC [56], 20% trichloroacetic acid (TCA, 50 μL) was added to RBC lysates (diluted 1/10), and the mixture was incubated (ice bath, 15 min) and centrifuged (15,000 g, 4 °C, 5 min). The supernatant was then discarded and 2,4-dinitrophenylhydrazine (10 mM in 2.5 N HCl) or HCL (2.5 N) was added to the tube of sample or blank solution, respectively. Following incubation (dark room, 1 h) and mixing (every 15 min), samples were centrifuged (15,000 g, 5 min, 4 °C), TCA (10%, 1 mL) was added to the supernatant, and the samples were then vortexed and centrifuged (15,000 g, 5 min, 4 °C). Subsequently, ethanol–ethyl acetate (1:1 *v*/*v*) was added to the new pellet, and the samples were centrifuged again (15,000 g, 4 °C, 5 min). The samples were then washed two more times, centrifuged once more, and urea (5 M, pH 2.3) was added to the pellet. The samples were then mixed and incubated (37 °C, 15 min). The samples were then centrifuged again (15,000 g, 4 °C, 3 min) and their absorbance was read spectrophotometrically at 375 nm. TBARS were analyzed as described [56]. Briefly, plasma samples were mixed with TCA (35%, 200 mM) and Tris–HCl (pH 7.4) and incubated (room temperature, 10 min). Na2SO4 (2M) and thiobarbituric acid (55 mM) were then added, and the resultant solution incubated (95 °C, 45 min). The samples were left to cool (5 min), had TCA (70%) added, were mixed and centrifuged (15,000 g, 3 min), and the absorbance of the supernatant was then read at 530 nm. For GSH measurement, RBC lysates were treated with 5% TCA, mixed with sodium potassium phosphate (67 mM, pH 8.0) and 5,5-dithio-bis-2 nitrobenzoate (1 mM), incubated (in the dark, 45 min, room temperature), and had their absorbance read spectrophotometrically at 412 nm [56]. For TAC analysis, sodium-potassium phosphate (10 mM, pH 7.4) and 2,2-diphenyl-1 picrylhydrazyl (0.1 mM) were added to serum samples, which were then incubated (in the dark, room temperature, 30 min), centrifuged (20,000 g, 3 min), and had their absorbance read spectrophotometrically at 520 nm [56]. Inter- and intra-assay coefficients in all assays performed ranged from 2.4 to 7.5% and from 3.4 to 8.1%, respectively.

### 2.9. Statistics

All data are presented as means ± SD. Data normality was verified using the Shapiro-Wilk test (*N* = 17/trial). Because we observed that our data sets in most of our variables did not follow normal distribution in at least one or two time-points, we applied non-parametric test. The Friedman analysis of variance by ranks test was used to determine the time-effect in each trial, accompanied by the Wilcoxon signed-rank test to perform pairwise comparisons. Differences across trials for all dependent variables were examined using the Kruskal-Wallis analysis of variance, and pairwise comparisons were performed using the Mann-Whitney U test. The level of statistical significance was set at *p* < 0.05. Effect sizes (ES) and confidence intervals (CI) were also calculated on results of all dependent variables using the Hedge’s g method, corrected for bias. Accordingly, effect size was interpreted as none, small, medium-sized, and large for values 0.00–0.19, 0.20–0.49, 0.50–0.79, and equal or >0.8, respectively. The SPSS software (IBM SPSS Statistics 20) was used for all analyses.

## 3. Results

**Figure 3:**
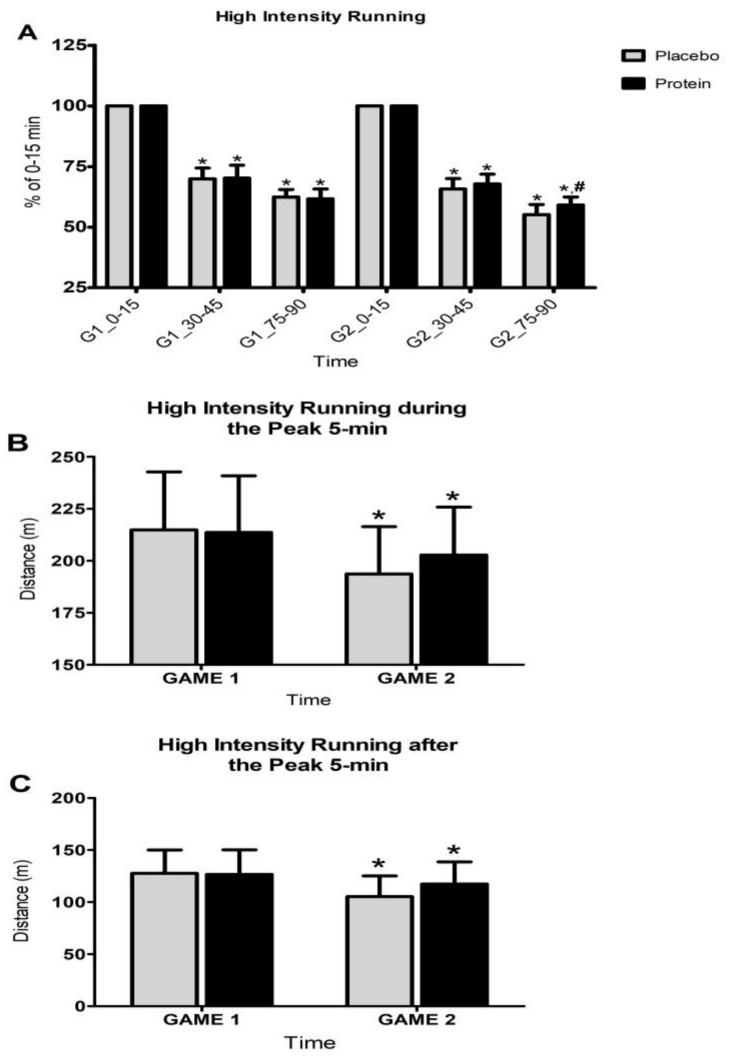
Changes in 15-min high-intensity running at the end of each half of each game compared to the first 15-period of the game (**A**), the high-intensity running distance during the peak 5-min (**B**), and the high-intensity running distance during the next 5-min after this peak 5-min period (**C**), during both games in each trial. G1, Game 1; G2, Game 2; * denotes a significant difference with baseline at *p* < 0.05; # denotes a significant difference between trials at *p* < 0.05.

**Figure 4:**
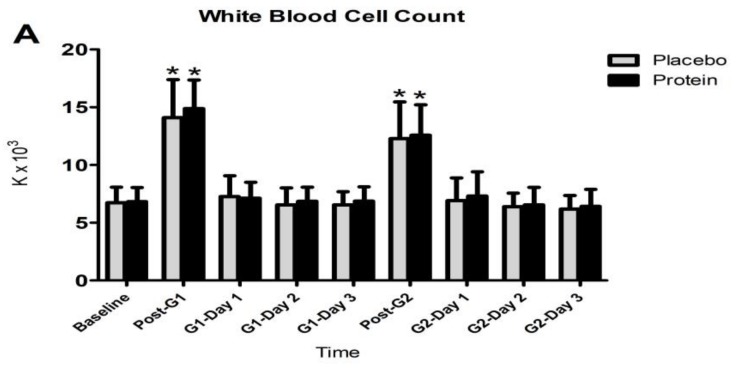

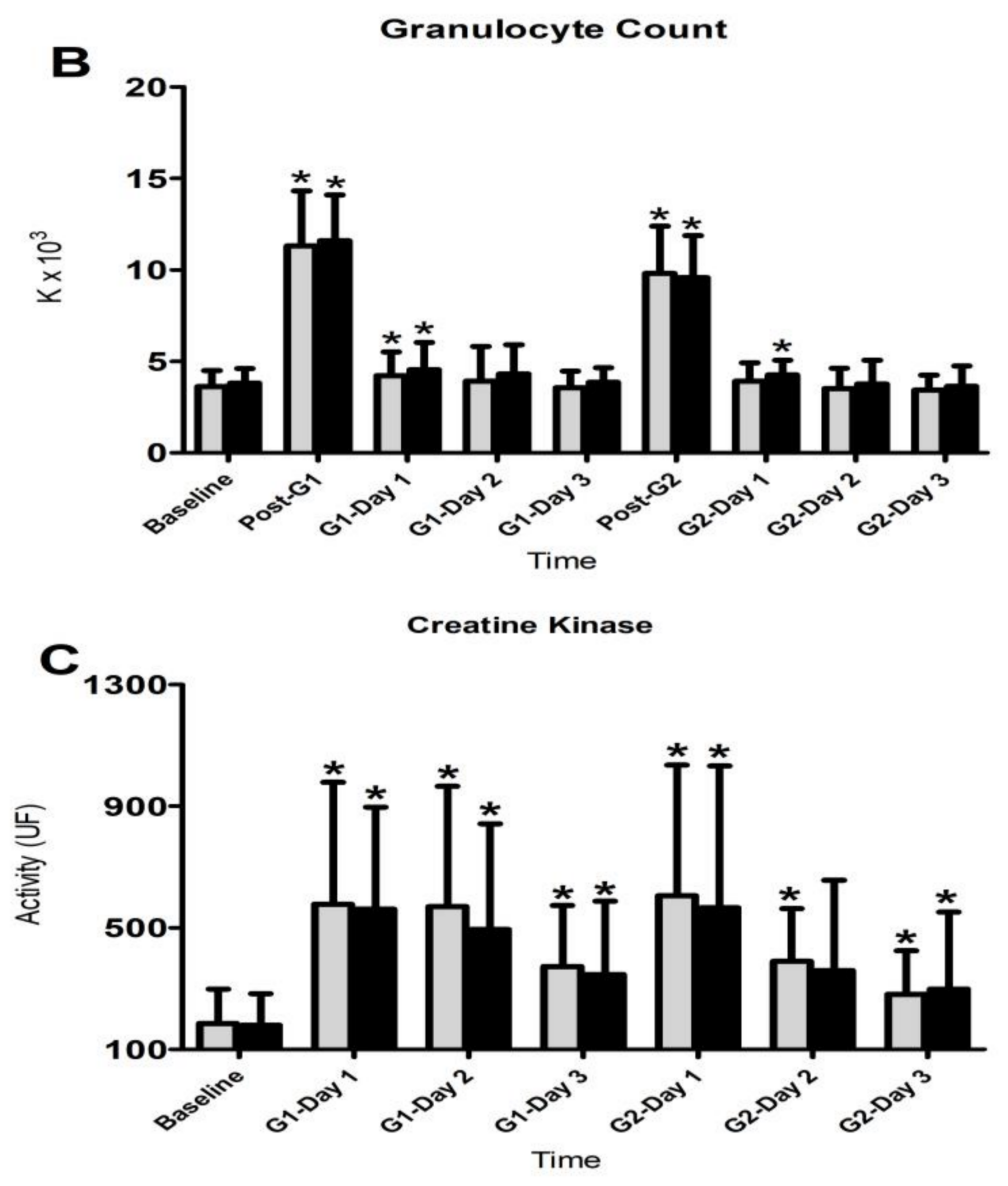
Changes in WBC count (**A**), granulocyte count (**B**), and creatine kinase activity (C). G1, game 1; G2, game 2; * denotes a significant difference with baseline at *p* < 0.05.

**Figure 5:**
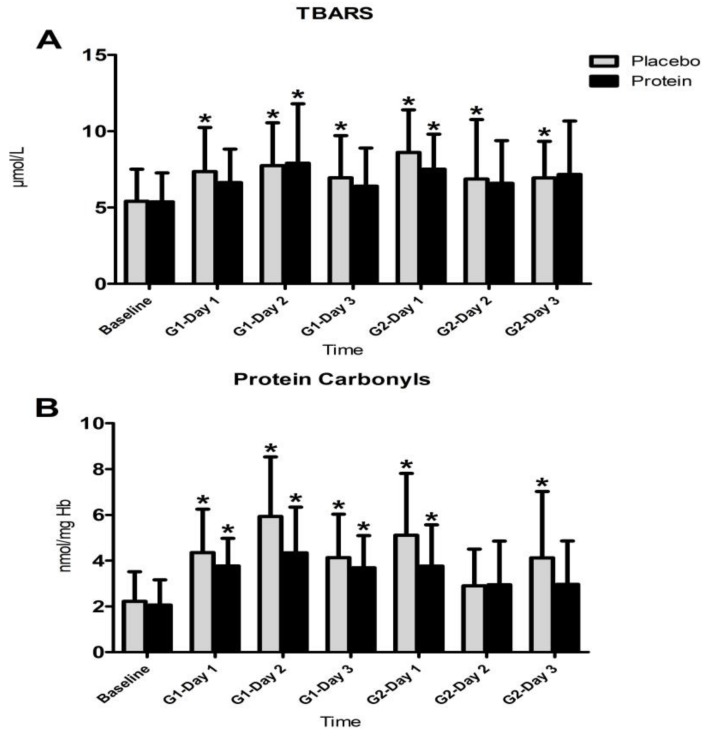
Changes in TBARS (**A**) and protein carbonyls (**B**). TBARS, thiobarbituric acid reactive substances; G1, game 1; G2, game 2; * denotes a significant difference with baseline at *p* < 0.05.

**Figure 6:**
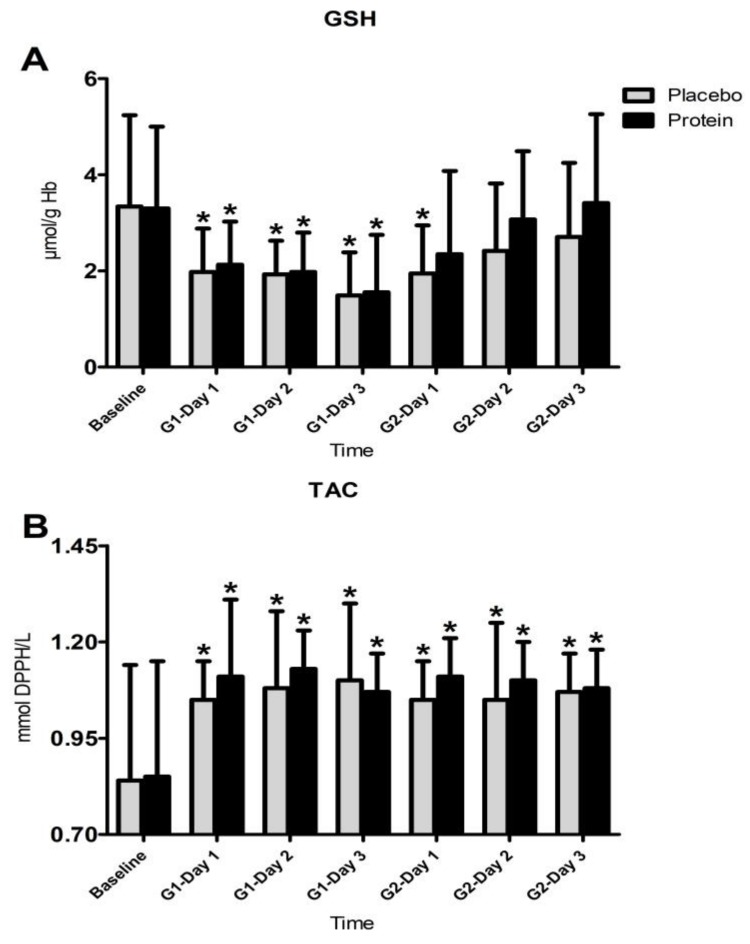
Changes in reduced glutathione (**A**) and total antioxidant activity (**B**). GSH, reduced glutathione; TAC, total antioxidant activity; G1, game 1; G2, game 2; * denotes a significant difference with baseline at *p* < 0.05.

Data from three players were excluded because they discontinued their participation due to injury (two during the first trial and one during the second trial). No between-trial differences were detected in respect to participants’ characteristics. Analysis of self-reported dietary data revealed that participants followed a similar dietary pattern throughout the study (Table 2). Baseline values (prior to each trial) of all variables examined were comparable (see Table 3, Table 4 and Table 5 and Figure 3, Figure 4, Figure 5 and Figure 6), suggesting that the wash-out period was effective to eliminate any systemic inflammation, and muscle damage manifestations developed in response to the first trial so that the two trials were performed under the same conditions. No adverse side effects were reported by the participants due to protein supplementation at the end of the study.

### 3.1. Game Activity Pattern

Table 3 presents changes in field activity variables obtained during the two games in response to PLA or PRO administration. Using Friedman analysis of variance by ranks test, a time effect was detected in PLA for average heart rate (x^2^ = 7.118, df = 1, P = 0.008), total distance covered during a match (x^2^ = 17.000, df = 1, P = 0.000), distance covered at a speed >14 km/h (x^2^ = 17.000, df = 1, P = 0.000), number of accelerations (x^2^ = 16.000, df = 1, P = 0.000), number of decelerations (x^2^ = 17.000, df = 1, P = 0.000), peak 5-min HIR distance (x^2^ = 9.941, df = 1, P = 0.002), and the next 5-min HIR distance (x^2^ = 17.000, df = 1, P = 0.000), but not for peak speed (x^2^ = 0.059, df = 1, P = 0.808). More specifically, in PLA the Wilcoxon test revealed a reduction from G1 to G2 for average heart rate (5.7%, PLA: z = 3.34, P = 0.001), total distance covered during a match (6%, z = 3.62, P = 0.000), distance covered at a speed > 14 km/h (11%, z = 3.62, P = 0.000), number of accelerations (1.9% z = 3.52, P = 0.000), number of decelerations (2.6%, z = 3.64, P = 0.000), peak 5-min HIR distance (10% z = 3.43, P = 0.001), and next 5-min HIR distance (17.6%, z = 3.62, P = 0.000). In PRO, Friedman analysis showed that distance covered at a speed >14 km/h (x^2^ = 9.941, df = 1, P = 0.002), number of accelerations (x^2^ = 12.250, df = 1, P = 0.000), number of decelerations (x^2^ = 9.000, df = 1, P = 0.003), peak 5-min HIR distance (x^2^ = 13.235, df = 1, P = 0.000), and the next 5-min HIR distance (x^2^ = 17.000, df = 1, P = 0.000), but not for average heart rate (x^2^ = 2.882, df = 1, P = 0.09), total distance covered during a match (x^2^ = 2.882, df = 1, P = 0.09), and peak speed (x^2^ = 0.0529, df = 1, P = 0.467). More specifically, in PRO the Wilcoxon test revealed a reduction from G1 to G2 for distance covered at a speed >14 km/h (5.7%, z = 3.24, P = 0.001), number of accelerations (1% z = 3.49, P = 0.000), number of decelerations (1.1%, z = 3.17, P = 0.002), peak 5-min HIR distance (5% z = 3.57, P = 0.000), and next 5-min HIR distance (7.2%, z = 3.62, P = 0.000). Kruskal-Wallis and Mann-Whitney analyses did not detect any statistically meaningful differences between trials despite the fact that the total distance and average heart rate remained unaffected in G2 compared to G1 in PRO but not in PLA, and PLA caused a far greater decline than PRO in all locomotor activity variables in G2 compared to G1 (distance covered with running at >14 km/h: 11% vs. 5.7%; number of accelerations: 2% vs. 1%; number of decelerations: 2.6% vs. 1%; peak 5-min: 10% vs. 5%; next 5-min: 18% vs. 7.2%).

Friedman analysis suggested that there were time-dependent changes both within each game (PLA: x^2^ = 76.025, df = 5, P = 0.000 for G1 and x^2^ = 77.509, df = 5, P = 0.000 for G2—PRO: x^2^ = 77.704, df = 5, P = 0.000 for G1 and x^2^ = 76.873, df = 5, P = 0.000 for G2) and in between games in both trials (PLA: x^2^ = 168.040, df = 11, P = 0.000, PRO: x^2^ = 167.834, df = 11, P = 0.000) for HIR performance during the last 15-min periods (Figure 3) of each half (0–15 min vs. 30–45 min and 0–15 min vs. 75–90 min). In other words, HIR performance demonstrated a marked decline at the end of each half (PLA-G1: z = 3.62, P = 0.000 for 0–15 min vs. 30–45 min and for 0–15 min vs. 75–90 min—PLA-G2: z = 3.62, P = 0.000 for 0–15 min vs. 30–45 min and for 0–15 min vs. 75–90 min—PRO-G1: z = 3.62, P = 0.000 for 0–15 min vs. 30–45 min and for 0–15 min vs. 75–90 min—PRO-G2: z = 3.62, P = 0.000 for 0–15 min vs. 30–45 min and z = 3.63, and P = 0.000 for 0–15 min vs. 75–90 min) and this decline deteriorated from G1 to G2 (PLA: z = 3.63, P = 0.000 for 0–15 min vs. 30–45 min and z = 3.52, P = 0.000 for 0–15 min vs. 75–90 min—PRO: z = 2.44, P = 0.015 for 0–15 min vs. 30–45 min and z = 3.65, and P = 0.008 for 0–15 min vs. 75–90 min) in both trials. When the two trials were compared, the Kruskal-Wallis and Mann-Whitney analyses showed that PRO induced a decline in 0–15 min vs. 75–90 min in G2 of smaller magnitude than that induced by PLA for the same time interval (U = 72.500, P = 0.012, ES = −1.01; CI: −1.72 to −0.29). Although the drop in HIR performance at the end of the first half in G2 (compared to G1) in PLA was almost twice as much as that in PRO (4.3% in PLA vs. 2.4% in PRO), no statistically meaningful differences were detected between trials. 

### 3.2. Activity Pattern During Training

Table 4 presents changes in field locomotor activity variables obtained during the four practices during the PLA and PRO trials. Using Friedman analysis of variance by ranks test, a time effect was detected in both PLA and PRO for average heart rate (PLA: x^2^ = 37.400, df = 3, P = 0.000; PRO: x^2^ = 33.994, df = 3, P = 0.000), total distance (PLA: x^2^ = 45.918, df = 3, P = 0.000; PRO: x^2^ = 44.435, df = 3, P = 0.000), and HIR (PLA: x^2^ = 43.376, df = 3, P = 0.000; PRO: x^2^ = 43.094, df = 3, P = 0.000). The Wilcoxon test revealed that after both games, the second practice was characterized by a greater average heart rate, total distance, and HIR compared to the first one in both trials. Kruskal-Wallis and Mann-Whitney analyses showed that there were no difference between trials in field locomotor variables during practices.

### 3.3. Performance

Table 5 presents changes observed in speed, CMJ, strength, and DOMS during both trials. The Friedman analysis of variance by ranks test revealed a time-dependent effect in both trials for 10-m sprint (PLA: x^2^ = 83.363, df = 6, P = 0.000; PRO: x^2^ = 90.580, df = 6, P = 0.000), 30-m sprint (PLA: x^2^ = 95.395, df = 6, P = 0.000; PRO: x^2^ = 97.084, df = 6, P = 0.000), CMJ (PLA: x^2^ = 59.674, df = 6, P = 0.000; PRO: x^2^ = 54.657, df = 6, P = 0.000), KE concentric strength of the dominant and non-dominant (PLA: x^2^ = 21.850, df = 6, P = 0.001—PRO: x^2^ = 12,874, df = 6, P = 0.045) limb, KF eccentric strength of the dominant (PLA: x^2^ = 27.446, df = 6, P = 0.000; PRO: x^2^ = 17.389, df = 6, P = 0.008) and non-dominant limb (PLA: x^2^ = 41.159, df = 6, P = 0.000; PRO: x^2^ = 49.538, df = 6, P = 0.000), DOMS of KE of the dominant (PLA: x^2^ = 51.150, df = 6, P = 0.000; PRO: x^2^ = 40.485, df = 6, P = 0.000) and non-dominant limb (PLA: x^2^ = 53.030, df = 6, P = 0.000; PRO: x^2^ = 46.889, df = 6, P = 0.000), and DOMS of KF of the dominant (PLA: x^2^ = 53.610, df = 6, P = 0.000; PRO: x^2^ = 57.512, df = 6, P = 0.000) and non-dominant limb (PLA: x^2^ = 58.248, df = 6, P = 0.000; PRO: x^2^ = 54.996, df = 6, P = 0.000). The Wilcoxon test revealed that 10-m sprint time (PLA: z = 3.10–3.62, P = 0.000–0.002—PRO: z = 3.13–3.62, P = 0.000–0.002), 30-m sprint time (PLA: z = 3.55–3.62, P = 0.000—PRO: z = 3.57–3.62, P = 0.000), CMJ (PLA: z = 3.43–3.62, P = 0.000–0.001—PRO: z = 3.46–3.62, P = 0.000–0.001), and DOMS of both KE (PLA: z = 3.31–3.65, P = 0.000–0.001—PRO: z = 3.20–3.66, P = 0.000–0.001) and KF (PLA: z = 3.53–3.71, P = 0.000—PRO: z = 3.32–3.74, P = 0.000–0.001) of both limbs deteriorated throughout recovery after both matches without any differences between trials noted by the Kruskal-Wallis and Mann-Whitney analysis.

KE concentric strength in PLA declined on days 1 (z = 2.368, P = 0.018) and 2 (z = 2.457, P = 0.014) and recovered on day 3 after G1 but declined throughout recovery after G2 (z = 2.083–2.415, P = 0.016–0.037). In contrast, in PRO, KE concentric strength declined on day 1 (z = 2.482, P = 0.013) after G1, recovered on days 2 and 3, and declined on days 1 (z = 2.864, P = 0.004) and 2 (z = 2.131, P = 0.033) but recovered on day 3 after G2. KF eccentric strength declined throughout recovery after G1 (PLA: z = 1.988–3.290, P = 0.001–0.047—PRO: z = 2.296–2.864, P = 0.004–0.22) in both trials, whereas after G2 it declined throughout recovery in PLA (z = 2.483–3.623, P = 0.000–0.013), but only during the first two days (z = 2.462–3.622, P = 0.000–0.014) in PRO. However, the Kruskal-Wallis and Mann-Whitney analysis did not detect any statistically meaningful differences between trials.

### 3.4. Inflammatory Responses

Figure 4 presents changes observed in WBC count granulocyte count and CK during both trials. The Friedman analysis of variance by ranks test revealed a time-dependent effect in both trials for WBC count (PLA: x^2^ = 86.826, df = 8, P = 0.000; PRO: x^2^ = 80.577, df = 8, P = 0.000), granulocyte count (PLA: x^2^ = 85.405, df = 8, P = 0.000; PRO: x^2^ = 82.291, df = 8, P = 0.000) and CK (PLA: x^2^ = 66.353, df = 6, P = 0.000; PRO: x^2^ = 70.588, df = 6, P = 0.000). The Wilcoxon test revealed that (i) the WBC count increased only immediately after each match in both trials (z = 3.62, P = 0.000 for both games and trials), (ii) the granulocyte count increased immediately during the first 24 h after each match (PLA: z = 2.10–3.62, P = 0.000–0.035—PRO: z = 2.73–3.62, P = 0.000–0.006) and subsided thereafter in both trials, and (iii) CK increased throughout recovery after both games in both trials (PLA: z = 2.95–3.62, P = 0.000–0.003—PRO: z = 2.72–3.62, P = 0.000–0.006). The Kruskal-Wallis and Mann-Whitney analysis indicated that there were no differences between trials in respect to changes in WBC, granulocyte count, and CK.

Figure 5 shows changes observed in oxidative stress markers, TBARS, and protein carbonyls during both trials. The Friedman analysis of variance by ranks test revealed a time-dependent effect in both trials for TBARS count (PLA: x^2^ = 14.471, df = 6, P = 0.025) but not for PRO (x^2^ = 6.939, df = 6, P = 0.327). The same analysis revealed a time-dependent change of protein carbonyls in both trials (PLA: x^2^ = 33.257, df = 6, P = 0.000—PRO: x^2^ = 23.378, df = 6, P = 0.001). The Wilcoxon test revealed that (i) TBARS in PLA increased throughout recovery after both games (z = 1.96–3.15, P = 0.002–0.049) but only one day after G2 in PRO (z = 2.76, P = 0.006) and (ii) protein carbonyls increased throughout recovery in PLA (z = 2.27–3.40, P = 0.001–0.023) but only until day 1 after G2 in PRO (z = 2.72–3.40, P = 0.001–0.006). Despite the fact that, according to the Wilcoxon test, TBARS and PC demonstrated a more short-lived rise in PRO compared to PLA, the Kruskal-Wallis and Mann-Whitney analysis did not reveal any differences between trials.

Figure 6 illustrates changes observed in antioxidant status markers, GSH, and TAC during both trials. The Friedman analysis of variance by ranks test revealed a time-dependent effect in both trials for GSH (PLA: x^2^ = 21.987, df = 6, P = 0.001—PRO: x^2^ = 23.395, df = 6, P = 0.001) and TAC (PLA: x^2^ = 18.927, df = 6, P = 0.004—PRO: x^2^ = 16.117, df = 6, P = 0.013). The Wilcoxon test showed that (i) GSH declined throughout recovery after G1 and for one day after G2 in PLA (z = 2.76–3.24, P = 0.001–0.006), whereas in PRO it declined only during recovery after G1 (z = 2.72–3.29, P = 0.001–0.006); and (ii) TAC increased throughout recovery in both trials (PLA: 2.30–3.62, P = 0.000–0.021—PRO: 2.46–3.46, P = 0.001–0.014). However, the Kruskal-Wallis and Mann-Whitney analysis did not detect any differences between trials.

## 4. Discussion

The main goal of this investigation was to examine whether football-specific and indirect performance measures are affected by an augmented total daily protein intake scheme during recovery from two games performed three days apart. Supplementary analyses of inflammatory markers were performed to characterize the overall muscle damage response. Our principal findings suggest that increased protein intake (i) may improve football locomotor activity during G2; (ii) seems to prevent a decline of KE and KF peak torque during recovery; and (iii) may promote antioxidant protection, especially after G2.

Our study utilized an experimental approach that has previously been applied to studying football-induced fatigue [19], inflammatory responses, and recovery kinetics [4,5,6,9,10]. This naturalistic approach may be more suitable for studying recovery kinetics in response to a football game than simulated protocols due to inclusion of football-specific activities [57]. In fact, the games in the present study were characterized by physiological responses, total distances covered by the players, and intensities similar to those observed in competitive games of elite football leagues as measured by GPS technology [7,58]. The pattern of performance decrement and the rise in inflammatory and muscle damage responses was similar to that previously reported after either a single [4,5,6,10,59] or multiple [9,30] football games.

An interesting result in the present study was the attenuation of the decline in locomotor activity and intensity (as evidenced by the lack of changes in total distance and average heart rate in G2 for PRO), as well as the increased fatigue resistance (as evidenced by the attenuated drop of HIR during the last 15 min of each half in G2) during G2 following protein supplementation. It must also be mentioned, however, that although statistical analyses did not reveal differences between trials, HIR and other field activity measures demonstrated a reduction in G2 that was almost half of that observed in PLA. This is the first study to use football game activity as a performance indicator to examine the effects of increased protein intake on performance of football players. It therefore appears that the PRO-trial improved some aspects of the locomotor activity profile during G2 performed only 3 days after G1. The only study that attempted to examine the effects of protein supplementation on game activity used a simulation protocol and not a real football game, and its results are similar [42]. In that study, investigators co-administered 2.5 mL/kg of either carbohydrates and protein (6% CHO plus 2% whey protein) or a carbohydrate (8% CHO) supplement to football and rugby athletes every 15 min during a modified Loughborough Intermittent Shuttle Test and monitored athletes’ activity pattern with GPS devices. They reported that co-ingestion of carbohydrates and PRO attenuated the drop in distance covered and maximal speed in the last 15-min of LIST. Similar results have been reported for basketball by Gentle et al. [60], who revealed that ingestion of a high-CHO/high-protein meal 90 min before a basketball game tended to reduce the drop in sprint performance and improve the free-throw success rate compared to the ingestion of only a high-CHO meal. However, in that study protein ingestion caused nausea during and after the game.

On the other hand, protein intake did not affect most indirect measures of performance either. Similar results have been reported by studies that used protein supplementation with football players. When Arent et al. [61] compared the effects of a more prolonged PRO supplementation protocol with isocaloric placebo supplementation in football players during pre-season training, they reported that PRO and PLA were equally effective at improving VO_2max_, velocity at lactate threshold, and endurance. Similarly, it was reported that consumption of either low-fat chocolate milk or an isocaloric high-carbohydrate supplement over a 4-day period did not affect agility, muscle power, strength, and fatigue resistance [40]. When semi-professional football players received a bolus of 500 mL of either plain water or semi-skimmed milk rich in whey protein and casein immediately after a muscle-damaging eccentric exercise protocol, the recovery of CMJ and reactive strength index were similar in the two trials, and there was only a tendency for a faster recovery of speed, agility, and passing performance in PRO compared to the water trial [62]. However, in that study, the two supplements compared were not isoenergetic, and the tendency to better performance may therefore be attributed to increased energy availability, since isoenergetic drinks were equally effective when administered before, during, and after a 90-min high-intensity, intermittent shuttle-running protocol [63]. When recreationally active, team sport athletes received either 1 L of a protein-CHO multi-ingredient, or a supplement with maltodextrin, or a placebo using repeated doses before, during, and 20 min after a 90-min intermittent repeated sprint protocol; performance during exercise or recovery was comparable between trials [43,44]. Consequently, it appears that protein supplementation, either alone or in combination with carbohydrates, does not affect most indirect markers of performance as it does with the activity pattern during a game. Future studies may therefore need to focus more on sport-specific field activity rather than on non-specific measures of performance. A potential explanation of such a dissociation between indirect performance tests and football-specific tests is that the former do not have such a large number of eccentric-type actions as those seen during actual football game or training activity (i.e., accelerations/decelerations, shooting, passing, high-volume intense running, tackling, jumping, etc.), and as such these tests may not be sensitive enough to detect performance changes related to these types of actions, because they provide a less challenging (sub-optimal) stimulus. However, protein supplementation did prevent a decline of KF and KE peak torque, especially following G2, a finding that may also explain the better HIR performance during G2 in PRO.

Participants in the PRO trial received an increased overall daily protein load (2.35 g/kg body mass or 2.62 g/kg fat-free mass) due to the high protein intake (1.49 g/kg body mass or 1.67 g/kg fat-free mass) during the 6-h post-game period, which is above the current guidelines for total daily protein intake for athletes [45,46]. This amount of post-exercise protein intake (80 g) using an intermediate feeding pattern is effective to stimulate protein synthesis [49,50]. The aim of this study was to provide isoenergetic protein and placebo drinks so that potential effects on recovery kinetics of performance markers could not be attributed to increased energy availability. Consequently, on game days total daily carbohydrate intake in PLA reached a level of 71% of total calories consumed (vs. 60% in PRO) (Table 2). This rise in total carbohydrate intake could not have influenced muscle bioenergetics and performance in this study since such an intake (71% vs. 55% in the control trial) failed to increase skeletal muscle glycogen resynthesis at rates over those measured in the controlled trial (5.9 trial vs. 6.0 mmol/kg dw/h in high carbohydrate and control trials, respectively, at 24 h post-game) after a football game [41]. Furthermore, increased carbohydrate availability in that study did not alter the concentrations of blood muscle damage makers, i.e., CK and myoglobin, during post-game recovery [41]. Ingestion of extra carbohydrate does not seem to affect performance, since consumption of a high-CHO/high-protein meal 90 min before a basketball game improved performance during the game, whereas consumption of only a high-CHO meal did not [60]. As such, there is no evidence to suggest that increased carbohydrate intake, as the one in the PLA trial of this study, may affect glycogen resynthesis, EIMD responses, and performance recovery.

The positive effects of protein supplementation on recovery kinetics of football-specific field performance and leg strength could potentially be explained by (i) improved skeletal muscle bioenergetics or (ii) improved neuromuscular performance or (iii) attenuation of the inflammatory response or (iv) improved skeletal muscle healing or (v) a combination of the above. Football-specific performance is heavily reliant on repeated sprint ability (RSA), which is related to phosphocreatine (PCr) availability, skeletal muscle glycogen stores, and mitochondrial adenosine triphosphate (ATP) production [19]. EIMD has been shown to promote metabolic acidosis of resting skeletal muscle and inhibition of mitochondrial function in exercising skeletal muscle [64]. Supplementation with branched-chain amino-acids (BCAAs) did not affect neuromuscular performance, concentration recovery rate of PCr, inorganic phosphate concentration, and pH under conditions of EIMD [65]. Although BCAAs and protein supplementation may elevate muscle protein synthesis, decrease protein oxidation, and promote mitochondrial biogenesis [66], proteins involved in mitochondrial respiration may be unresponsive to whey protein feeding following intense exercise [38]. Protein supplementation did not interfere with the exercise-induced upregulation of adenosine monophosphate activated protein kinase (AMPK), which is considered the principal controller of mitochondrial biogenesis and metabolism [67]. Moreover, it appears that glycogen resynthesis remains unaffected by protein supplementation after a football game [41] or intense continuous exercise [67]. Therefore, the available evidence does not support an effect of protein supplementation on skeletal muscle bioenergetics during recovery from intense damaging exercise. As such, the improvement in field performance in this study may not be related to altered skeletal muscle bioenergetics.

Protein supplementation may induce improved neuromuscular performance. There are numerous reports that various forms of protein supplementation (e.g., fast digestible soluble milk protein, milk protein concentrate, oat protein, and whey protein isolate) may prevent or attenuate skeletal muscle performance deterioration following an exercise damaging protocol or even increase skeletal muscle fatigue resistance [48,68,69,70,71]. These effects may be mediated by an upregulation of skeletal muscle carnosine content [72]. Although there is considerable evidence of increased neuromuscular performance and fatigue resistance following protein supplementation during recovery following a damaging exercise protocol, electromyogram was unresponsive to beta-alanine supplementation after high-intensity interval exercise [73]. Improved neuromuscular function and fatigue resistance may therefore offer a plausible explanation for the results observed in this study.

Protein supplementation attenuated the CK rise elicited by damaging exercise in most studies [40,43,60,61]. However, three other studies showed no effect [44,62,63], and one reported a rise in CK response during recovery in PRO compared to PLA administration [41]. In this study, CK elevation was attenuated in PRO but with a small ES. As such, no formal conclusion can be reached regarding the effects of protein supplementation on muscle damage based on CK responses. Protein-induced attenuation of the inflammatory response may offer a possible explanation for the improvement in recovery kinetics of football-specific performance in PRO. Findings from previous studies have been highly inconclusive reporting both attenuation [70,74,75,76] and no change [63,77,78,79,80] of muscle damage markers in response to various exercise protocols. The mechanisms linking protein intake with muscle soreness and CK responses are largely unknown, and only speculations can be made. The positive effects of increased protein intake on performance recovery may be related to anti-inflammatory actions rather than EIMD-related mechanisms. In fact, it has been shown that augmented protein intake induces an attenuation of C reactive protein and myeloperoxidase elevation following a muscle damaging protocol [75]. Administration of protein and/or BCAAs may also interfere with the acute phase response of the immune system following damaging exercise [81,82], probably via the mammalian target of rapamycin (mTOR) intracellular signaling pathway [83] or through the modulation of neutrophils’ oxidative burst [84]. Leukocytes, initially neutrophils and then macrophages, reach the injured tissue following damaging exercise to mediate not only the removal of cellular debris but also to control the repair of damaged myofibre [23]. Sulphur-containing amino acids may modulate cellular redox status and thus suppress the synthesis and release of pro-inflammatory cytokines (e.g., IL-1, IL-6) that contribute to the mobilization of immune cells and as such reduce the post-exercise leukocyte counts [85]. However, no differences were noted between trials in WBC count following both games. Similar results have been reported for the immune system in response to co-ingestion of carbohydrates and protein by football players after a sprint protocol [44,86]. The lack of response by leukocytes to protein supplementation after intense exercise may be related to the absence of changes in cytokines involved in the mediation of the immune response, i.e., IL-1 and IL-6 [44,85]. Nevertheless, Nelson et al. [87] reported a small attenuation of leukocyte counts by protein feeding during intense training. Differences in exercise modalities, overload characteristics, and composition of protein supplements may have contributed to this discrepancy.

Protein’s anti-inflammatory actions may include antioxidant actions, since TBARS, protein carbonyls rise, and GSH decline were more prolonged in PLA compared to PRO. Oxidative burst of leukocytes is the main source of reactive oxygen species (ROS) generation during recovery after EIMD [13]. A reduction of myeloperoxidase activity in response to protein feeding under conditions of EIMD further supports this possibility [75]. This may be mediated by a protein-induced increase of antioxidant reserves. Leucine-protein supplementation mitigates the superoxide release by neutrophils following intense exercise [87] and upregulates GSH reserves in leukocytes [88]. However, in this study, WBC and granulocytes counts increased similarly in PLA and PRO following both games. Previous findings suggested that protein supplementation may not alter ROS production by neutrophils after very intense exercise despite a positive effect on their survival rate [89]. However, in this study a time effect was revealed by statistical analysis, i.e., protein oxidation (protein carbonyls) and lipid peroxidation (TBARS) demonstrated more prolonged responses in PLA compared to PRO. Specifically, TBARS in PLA remained above baseline values throughout recovery after both games but only for one day after G2 in PRO. Similarly, protein carbonyls remained elevated throughout recovery in PLA but only until day 1 after G2 in PRO. Although direct statistical comparison between trials revealed no statistically meaningful differences, the rise of oxidative stress markers in PRO exhibited a faster recovery rate compared to PLA, especially after G2. Although such an antioxidant effect of protein ingestion following football activity was also reported by Arent et al. [61], others have reported no changes of ROS generation and/or oxidative stress markers in response to protein feeding [78,87]. Therefore, results of this study suggest that increased protein feeding may reduce the time of elevation of oxidative stress markers, which could potentially explain the positive effect seen on acceleration of performance recovery following repeated football games. This effect is probably mediated by a depletion of antioxidant reserves of smaller magnitude, since protein feeding resulted in a more prolonged reduction of GSH in PLA (throughout recovery after G1 and for one day after G2) compared to PRO (during recovery after G1), despite the fact that no statistically meaningful differences were detected between trials.

Accelerated skeletal muscle healing may facilitate performance recovery following intense exercise protocols. Satellite cells are pivotal for skeletal muscle regeneration following trauma [90]. Although phosphorylation of proteins involved in intracellular anabolic (mTOR, p70S6K, rpS6) and catabolic signaling (FOXO1, FOXO3) were unresponsive to supplementation with animal- and plant-derived protein [77], satellite cell content and activation was markedly upregulated with increased protein feeding following concentric and eccentric exercise [80,91,92]. These observations are in line with reports of an improved overnight whole-body nitrogen balance in response to protein ingestion when compared to controlled feeding under conditions of EIMD [78]. These observations were corroborated by other studies that reported a reduction in the inflammatory response, an upregulation of proteasome activity, an attenuation of protein breakdown, and a rise of proliferating satellite cells in skeletal muscle by leucine ingestion during recovery from muscle trauma induced by cryolesion or acute resistance exercise [93,94]. Participants in this study were provided with ~25–30 g and ~5 g of BCAAs on game and training days, respectively. BCAAs, particularly leucine, have been shown to provide a potent stimulus for enhanced ribosomic protein translation and attenuated proteolysis [48,49]. Moreover, BCAAs may be transaminated, under pro-inflammatory conditions, to glutamate, thereby enhancing glutamine synthesis, which is a substrate highly used by pro-inflammatory immune cells such as macrophages. Thus, BCAAs may contribute to remodeling of injured myofiber by upregulating protein synthesis and reducing its degradation on one hand and modulate the inflammatory response on the other [95]. These studies suggest that the acceleration of football-specific and strength performance observed in this study may be related to the promotion of a more anabolic environment through the upregulation of satellite cell numbers and activation, increased protein synthesis, reduced protein degradation, and modulation of the inflammatory response.

## 5. Conclusions

It has been shown that football activity augments daily protein requirements above the safe RDA levels currently recommended [37]. Results of the present study indicate that protein supplementation may facilitate recovery of football-specific performance during an in-season microcycle consisting of two games performed three days apart and daily practices. This effect is probably attributable to an attenuation of neuromuscular fatigue, a reduced inflammatory response, and the accelerated regeneration of skeletal muscle. Future research should further explore (i) the effects of increased protein intake on performance recovery in football by investigating the dose-response relationship and examining the value of protein source for optimal recovery and (ii) if the source of protein in the form of food would be able to mimic the results seen in this study.

## Figures and Tables

**Table 1:** Participants’ characteristics at baseline.

	Male (*n* = 20)
Age (years)	20.6 ± 1.1
Height (cm)	179.1 ± 4.6
Weight (kg)	77.6 ± 5.8
BMI (kg/m^2^)	24.0 ± 1.0
Body fat (%)	9.9 ± 2.2
RMR (kj/day)	6099.8 ± 641.40
VO_2max_ (mL/kg/min)	57.7 ± 3.4
Maximal heart rate (bpm)	196.8 ± 6.3
Yo-Yo IE2 (m)	2890.5 ± 303.7
Yo-Yo IR2 (m)	1638.44 ± 153.8

BMI, body mass index; RMR, resting metabolic rate; VO_2max_, maximal oxygen consumption, bpm, beats per minute; IE, intermittent endurance; IR, intermittent recovery.

**Table 2:** Participants’ dietary energy intake and antioxidant profiles during the study.

	PLA			PRO		
	Diet	Placebo	Total	Diet	Supplement	Total
**Daily Energy Intake (kJ/kg BM/day)**						
Adaptive period	119.1 ± 8.1	N/A	119.1 ± 8.1	120.1 ± 8.0	N/A	120.1 ± 8.0
Game days	191.1 ± 8.2	22.8 ± 0.0	213.9 ± 8.2	191.2 ± 7.4	23.0 ± 0.0	214.2 ± 7.4
Training days	152.4 ± 6.4	5.2 ± 0.0	157.6 ± 6.4	152.0 ± 6.3	5.25 ± 0.0	157.2 ± 6.3
**Carbohydrate intake**						
Adaptive period (g/kg BM)	3.72 ± 0.2	N/A	3.72 ± 0.2	3.78 ± 0.2	N/A	3.78 ± 0.2
Adaptive period (% of total intake)	52.2 ± 2.4			52.6 ± 2.1		
Game days: pre-game period (g/kg BM)	2.27 ± 0.4	N/A	2.27 ± 0.4	2.28 ± 0.4	N/A	2.28 ± 0.4
Game days: post-game period (g/kg BM)	4.46 ± 0.2	1.37 ± 0.0	5.83 ± 0.2	4.50 ± 0.1	0.19 ± 0.0	4.69 ± 0.1*
Game days, total (total g/kg BM)	6.74 ± 0.3	1.37 ± 0.0	8.11 ± 0.3	6.78 ± 0.4	0.19 ± 0.0	6.97 ± 0.4*
Game days (% of total intake)	71.0 ± 1.3			61.0 ± 1.4		
Training days (g/kg BM)	5.11 ± 0.3	0.31 ± 0.0	5.44 ± 0.3	5.17 ± 0.2	0.05 ± 0.0	5.22 ± 0.2
Training days (% of total intake)	59.7 ± 3.8			57.4 ± 3.9		
**Protein intake**						
Adaptive period (g/kg BM)	1.03 ± 0.05	N/A	1.03 ± 0.05	1.05 ± 0.04	N/A	1.05 ± 0.04
Adaptive period (g/kg FFM)	1.15 ± 0.06	N/A	1.15 ± 0.06	1.17 ± 0.05	N/A	1.17 ± 0.05
Adaptive period (% of total intake)	14.5 ± 1.2			14.6 ± 1.1		
Game days: pre-game period (g/kg BM)	0.85 ± 0.05	N/A	0.85 ± 0.05	0.86 ± 0.05	N/A	0.86 ± 0.05
Game days: pre-game period (g/kg FFM)	0.90 ± 0.2	N/A	0.90 ± 0.2	0.95 ± 0.05	N/A	0.95 ± 0.05
Game days: post-game recovery (g/kg BM)	0.47 ± 0.04	0.0 ± 0.0	0.47 ± 0.04	0.46 ± 0.04	1.03 ± 0.0	1.49 ± 0.04 *
Game days: post-game recovery (g/kg FFM)	0.53 ± 0.05	0.0 ± 0.0	0.53 ± 0.05	0.52 ± 0.05	1.15 ± 0.0	1.67 ± 0.05
Game days, total (g/kg BM)	1.33 ± 0.1	0.0 ± 0.0	1.33 ± 0.1	1.32 ± 0.07	1.03 ± 0.0	2.35 ± 0.07 *
Game days (g/kg FFM)	1.48 ± 0.1	0.0 ± 0.0	1.48 ± 0.1	1.47 ± 0.07	1.15 ± 0.0	2.62 ± 0.07 *
Game days (% of total intake)	13.0 ± 1.1			22.9 ± 0.4		
Training days (g/kg BM)	1.21 ± 0.04	0.0 ± 0.0	1.21 ± 0.04	1.22 ± 0.04	0.26 ± 0.0	1.48 ± 0.04
Training days (g/kg FFM)	1.35 ± 0.06	0.0 ± 0.0	1.35 ± 0.06	1.36 ± 0.06	0.29 ± 0.0	1.65 ± 0.06
Training days (% of total intake)	14.8 ± 0.9			18.2 ± 1.0		
**Fat intake (g/kg)**						
Adaptive period (g/kg BM)	1.05 ± 0.1	N/A	1.05 ± 0.1	1.06 ± 0.1	N/A	1.06 ± 0.1
Game days (g/kg BM)	1.50 ± 0.1	0.0 ± 0.0	1.50 ± 0.1	1.49 ± 0.1	0.06 ± 0.0	1.55 ± 0.1
Game days (% of total intake)	29.6 ± 1.7			30.5 ± 1.5		
**Selenium (μg/day)**						
Adaptive period	43.9 ± 9.6	0.0 ± 0.0	43.9 ± 9.6	44.5 ± 10.3	0.0 ± 0.0	44.5 ± 10.3
Game days	48.8 ± 12.4	0.0 ± 0.0	47.4 ± 12.4	46.2 ± 9.7	0.0 ± 0.0	46.2 ± 9.7
Training days	46.7 ± 11.3	0.0 ± 0.0	46.7 ± 11.3	45.9 ± 10.9	0.0 ± 0.0	45.9 ± 10.9
**Zinc (mg/day)**						
Adaptive period	10.7 ± 2.8	0.0 ± 0.0	10.7 ± 2.8	10.9 ± 3.3	0.0 ± 0.0	10.9 ± 3.3
Game days	11.1 ± 3.2	0.0 ± 0.0	11.1 ± 3.2	11.8 ± 3.6	0.0 ± 0.0	11.8 ± 3.6
Training days	11.0 ± 2.9	0.0 ± 0.0	11.0 ± 2.9	11.5 ± 3.6	0.0 ± 0.0	11.5 ± 3.6
**Vitamin C (mg/day)**						
Adaptive period	123.6 ± 9.4	0.0 ± 0.0	123.6 ± 9.4	121.9 ± 9.1	0.0 ± 0.0	121.9 ± 9.1
Game days	128.3 ± 11.8	0.0 ± 0.0	128.8 ± 11.8	124.7± 8.1	0.0 ± 0.0	124.7± 8.1
Training days	126.7 ± 11.2	0.0 ± 0.0	126.7 ± 11.2	125.5 ± 11.0	0.0 ± 0.0	125.5 ± 11.0
**Vitamin E (mg/day, α-TE)**						
Adaptive period	8.7 ± 4.1	0.0 ± 0.0	8.7 ± 4.1	8.9 ± 4.2	0.0 ± 0.0	8.9 ± 4.2
Game days	9.1 ± 4.5	0.0 ± 0.0	9.1 ± 4.5	9.3 ± 4.7	0.0 ± 0.0	9.3 ± 4.7
Training days	8.8 ± 4.4	0.0 ± 0.0	8.8 ± 4.4	9.2 ± 4.3	0.0 ± 0.0	9.2 ± 4.3

PLA, the placebo trial; PRO, the protein trial; BM, body mass; FFM, fat-free mass; * statistical differences detected between trials at *p* < 0.05.

**Table 3:** Changes in game field activity in response to protein and placebo consumption.

	Game 1	Game 2
Average HR (beats/min)		
PLA	166.9 ± 6.6	157.4 ± 8.51 ^1^
PRO	167.2 ± 6.9	161.5 ± 8.81
Total distance (m)		
PLA	10,048.1 ± 748.5	9448.8 ± 545.2 ^1^
PRO	10,032.2 ± 768.6	9756.6 ± 553.8
Distance at >14 km/h		
PLA	1890.5 ± 245.4	1683.8 ± 232,9 ^1^
PRO	1909.0 ± 240.1	1800.3 ± 235.7 ^1^
Peak speed (km/h)		
PLA	27.8 ± 2.6	27.4 ± 1.7
PRO	27.1 ± 3.0	27.0 ± 2.4
Accelerations (no)		
PLA	268.0 ± 18.9	262.9 ± 19.7 ^1^
PRO	267.9 ± 20.5	265.0 ± 20.4 ^1^
Decelerations (no)		
PLA	266.8 ± 18.1	259.8 ± 19.2 ^1^
PRO	265.3 ± 20.3	262.5 ± 20.0 ^1^

PLA, the placebo trial; PRO, the protein trial; ^1^ significant difference between games; ^2^ significant difference between PRO and PLA.

**Table 4:** Changes in practice field activity in response to protein and placebo consumption.

	Game 1-D1	Game 1-D2	Game 2-D1	Game 2-D2
Average HR (beats/min)				
PLA	125.5 ± 8.2 ^1^	132.8 ± 6.8 ^1^	123.8 ± 5.9 ^1^	154.2 ± 5.9 ^1^
PRO	126.4 ± 10.2 ^1^	131.9 ± 9.3 ^1^	124.4 ± 3.9 ^1^	161.0 ± 6.7 ^1^
Total distance (m)				
PLA	2543.1 ± 776.3 ^1^	4414.0 ± 485.8 ^1^	2341.1 ± 392.9 ^1^	5366.4 ± 367.4 ^1^
PRO	2562.8 ± 883.6 ^1^	4337.4 ± 446.9 ^1^	2385.2 ± 342.8 ^1^	5565.2 ± 383.3 ^1^
High-intensity running (m)				
PLA	172.4 ± 26.3 ^1^	947.7 ± 93.2 ^1^	153.8 ± 26.4 ^1^	1223.1 ± 379.8 ^1^
PRO	170.8 ± 28.1 ^1^	941.2 ± 116.5 ^1^	155.8 ± 29.7 ^1^	1343.8 ± 366.6 ^1^

PLA, the placebo trial; PRO, the protein trial; D1, practice performed 24 h after each game; D2, practice performed 48 h after each game; m, meters; HR, heart rate; MHR, maximal heart rate; ^1^ significant difference between D1 and D2 for each game at *p* < 0.05.

**Table 5:** Changes in performance measures in response to protein and placebo consumption.

	Baseline	Game 1-D1	Game 1-D2	Game 1-D3	Game 2-D1	Game 2-D2	Game 2-D3
**10-m sprint (s)**							
PLA	1.81 ± 0.06	2.00 ± 0.07 ^1^	1.93 ± 0.07 ^1^	1.87 ± 0.08 ^1^	2.09 ± 0.13 ^1^	1.97 ± 0.06 ^1^	1.88 ± 0.06 ^1^
PRO	1.80 ± 0.06	2.01 ± 0.07 ^1^	1.93 ± 0.06 ^1,2^	1.86 ± 0.07 ^1^	2.09 ± 0.08 ^1^	1.98 ± 0.06 ^1,2^	1.88 ± 0.08 ^1^
**30-m sprint (s)**							
PLA	4.18 ± 0.18	4.70 ± 0.21 ^1^	4.54 ± 0.18 ^1^	4.38 ± 0.14 ^1^	4.90 ± 0.28 ^1^	4.62 ± 0.22 ^1^	4.48 ± 0.2 ^1^
PRO	4.19 ± 0.16	4.67 ± 0.19 ^1^	4.53 ± 0.17 ^1^	4.38 ± 0.13 ^1^	4.89 ± 0.26 ^1^	4.62 ± 0.19 ^1^	4.45 ± 0.15 ^1^
**CMJ (cm)**							
PLA	48.6 ± 4.3	43.9 ± 3.9 ^1^	45.5 ± 4.5 ^1^	47.1 ± 4.3 ^1^	43.6 ± 4.2 ^1^	43.9 ± 3.4 ^1^	45.1 ± 3.0 ^1^
PRO	48.7 ± 4.4	44.2 ± 4.5 ^1^	45.7 ± 3.4 ^1^	47.2 ± 4.5 ^1^	44.5 ± 3.5 ^1^	44.6 ± 3.3 ^1^	45.8 ± 3.1 ^1^
**KE concentric strength/Dominant limb (Nm/kg)**							
PLA	3.31 ± 0.7	3.05 ± 0.6 ^1^	3.13 ± 0.6 ^1^	3.20 ± 0.6	3.00 ± 0.6 ^1^	3.02 ± 0.6 ^1^	3.12 ± 0.7 ^1^
PRO	3.33 ± 0.7	3.11 ± 0.7 ^1^	3.15 ± 0.7	3.22 ± 0.7	3.05 ± 0.6 ^1^	3.12 ± 0.7 ^1^	3.17 ± 0.6
**KE concentric strength/Non-Dominant limb (Nm/kg)**							
PLA	3.34 ± 0.7	3.10 ± 0.6 ^1^	3.15 ± 0.7 ^1^	3.22 ± 0.7	3.12 ± 0.6 ^1^	3.14 ± 0.6	3.23 ± 0.7 ^1^
PRO	3.33 ± 0.7	3.11 ± 0.6 ^1^	3.16 ± 0.6	3.20 ± 0.7	3.12 ± 0.6 ^1^	3.13 ± 0.7 ^1^	3.21 ± 0.6
**KF eccentric strength/Dominant limb (Nm/kg)**							
PLA	3.20 ± 0.5	2.66 ± 0.4 ^1^	2.91 ± 0.4 ^1^	2.92 ± 0.3 ^1^	2.72 ± 0.3 ^1^	2.73 ± 0.4 ^1^	2.96 ± 0.4 ^1^
PRO	3.17 ± 0.5	2.80 ± 0.3 ^1^	2.82 ± 0.4 ^1^	2.93 ± 0.3 ^1^	2.75 ± 0.4 ^1^	2.78 ± 0.4 ^1^	3.02 ± 0.4
**KF eccentric strength/non-dominant limb (Nm/kg)**							
PLA	3.23 ± 0.5	2.69 ± 0.5 ^1^	2.90 ± 0.4 ^1^	3.03 ± 0.5 ^1^	2.49 ± 0.6 ^1^	2.73 ± 0.4 ^1^	2.98 ± 0.4 ^1^
PRO	3.20 ± 0.4	2.73 ± 0.3 ^1^	2.89 ± 0.4 ^1^	3.04 ± 0.3 ^1^	2.53 ± 0.5 ^1^	2.73 ± 0.5 ^1^	3.02 ± 0.3
**DOMS, KE/Dominant limb**							
PLA	1.0 ± 0.0	4.3 ± 1.7 ^1^	3.6 ± 1.5 ^1^	3.3 ± 1.2 ^1^	4.2 ± 1.3 ^1^	3.1 ± 1.4 ^1^	3.0 ± 1.3 ^1^
PRO	1.0 ± 0.0	3.2 ± 1.6 ^1^	3.3 ± 1.2 ^1^	2.9 ± 1.2 ^1^	3.7 ± 1.1 ^1^	3.2 ± 1.6 ^1^	2.9 ± 1.2 ^1^
**DOMS, KE/non-dominant limb**							
PLA	1.0 ± 0.0	4.5 ± 1.3 ^1^	3.8 ± 1.8 ^1^	3.7 ± 1.4 ^1^	4.2 ± 1.6 ^1^	3.4 ± 1.9 ^1^	2.9 ± 1.3 ^1^
PRO	1.0 ± 0.0	3.6 ± 2.0 ^1^	3.7 ± 1.7 ^1^	3.5 ± 2.0 ^1^	4.5 ± 1.7 ^1^	3.6 ± 1.9 ^1^^,5^	3.0 ± 1.3 ^1^
**DOMS, KF/Dominant limb**							
PLA	1.0 ± 0.0	4.7 ± 1.5 ^1^	4.3 ± 1.3 ^1^	3.7 ± 1.2 ^1^	4.6 ± 1.1 ^1^	4.4 ± 1.3 ^1^	3.7 ± 1.2 ^1^
PRO	1.0 ± 0.0	3.8 ± 1.7 ^1^	4.0 ± 1.4 ^1^	3.4 ± 1.2 ^1^	4.7 ± 1.1 ^1^	4.5 ± 1.2 ^1^	3.5 ± 1.2 ^1^
**DOMS, KF/non-dominant limb**							
PLA	1.0 ± 0.0 ^1^	4.4 ± 1.4 ^1^	4.7 ± 1.1 ^1^	3.8 ± 1.1 ^1^	4.8 ± 1.0 ^1^	4.1 ± 1.2 ^1^	4.0 ± 0.7 ^1^
PRO	1.0 ± 0.0	3.7 ± 1.6 ^1^	4.1 ± 1.2 ^1^	3.7 ± 1.0 ^1^	4.7 ± 1.1 ^1^	4.1 ± 1.0 ^1^	3.8 ± 0.7 ^1^

Baseline and measurements obtained before Game 1; D1, 24 h after each game; D2, 48 h after each game; D3, 72 h after each game; PLA, the placebo trial; PRO, the protein trial; s, seconds; DOMS, delayed onset of muscle soreness; KE, knee extensors; DL, dominant limb; NDL, non-dominant limb; KF, knee flexors; ^1^ significantly different from baseline at *p* < 0.05.
